# Natural Recovery in Trichotillomania

**DOI:** 10.1177/00048674211066004

**Published:** 2021-12-14

**Authors:** Jon E. Grant, Samuel R. Chamberlain

**Affiliations:** a Department of Psychiatry & Behavioral Neuroscience, University of Chicago, Chicago, IL, USA; b Department of Psychiatry, University of Southampton, UK

**Keywords:** trichotillomania, natural recovery, outcomes, comorbidity

## Abstract

**Objectives:**

Trichotillomania is characterized by repetitive pulling out of one’s hair, leading to distress and/or functional impairment. Long considered a chronic condition if left untreated (albeit with fluctuating intensity), there have been intimations that the disorder may be of limited duration in some people.

**Methods:**

A sample of 10,169 adults, aged 18-69 years, representative of the general US population, were recruited and screened for current and lifetime trichotillomania. Potential differences in demographic and clinical variables and lifetime comorbidities, between those with natural recovery from trichotillomania, and those with current trichotillomania, were identified using analysis of variance or Likelihood-Ratio chi-square tests as appropriate. Additional analyses using binary logistic regression were used to control for potential confounding differences between the groups initially identified.

**Results:**

24.9% of the entire sample of people with lifetime trichotillomania reported that they no longer had symptoms of trichotillomania and had never received therapy or medication treatment for it (i.e. they experienced natural recovery). Those who experienced natural recovery did not differ from those with current trichotillomania in terms of demographic or clinical characteristics, except that they were currently older. Natural recovery was associated with significantly lower rates of related comorbidities: obsessive-compulsive disorder (OCD), attention-deficit hyperactivity disorder (ADHD), panic disorder, skin picking disorder, and tic disorder.

**Discussion:**

These findings from the first epidemiology study examining natural recovery in trichotillomania highlight the importance of screening for and treating such comorbidities in patients with trichotillomania, in order to maximize chance of clinical recovery.

## Introduction

Trichotillomania (hair-pulling disorder) is characterized by recurrent pulling out of one’s own hair, leading to hair loss and oftentimes functional impairment (American Psychiatric Association, 2013). Although examined in the medical literature for decades (Grant and Chamberlain, 2016), the course of trichotillomania has been the subject of much debate. Long considered a chronic condition if left untreated (albeit with fluctuating intensity) (Christenson et al., 1991a, 1991b), there have been intimations that the disorder may be of limited duration in some people. For example, in the case of childhood-onset trichotillomania (i.e. those whose hair pulling begins before the age of 5 years), many simply stop pulling over time (Swedo and Leonard, 1992). Other research suggests that trichotillomania may become chronic in people who have pulled for more than 6 months (Chang et al., 1992). Research on the clinical characteristics, neurobiology, and treatment of trichotillomania has expanded over the last thirty years (Grant and Chamberlain, 2016), but research on the natural course of the disorder and any putative predictors of subsequent recovery is scant. Such research is needed in light of findings indicating that trichotillomania results in a heavy psychosocial burden of suffering (Grant et al., 2016; Franklin et al., 2008; Houghton et al., 2016; Tung et al., 2015).

One area of trichotillomania research in need of further elaboration is that of natural recovery (i.e. having met diagnostic criteria for the disorder in the past but not meeting any diagnostic criteria for the disorder for the past 12 months and attaining this achievement without any formal psychological or pharmacological interventions). Our definition is consistent with the one proposed by Slutske (2006), although others have defined the term as achieving remission for the short term (i.e. 2-3 months) from a disorder but still possibly having some mild symptoms (Mekonen et al., 2021). When we look to other mental health disorders, we find some limited understanding of this phenomenon. In the case of major depressive disorder, a recent metaanalysis found a pooled estimate of 12.5% of people with untreated depression achieved remission within 12 weeks (Mekonen et al., 2021). In the area of obsessive compulsive disorder, a disorder with some phenomenological similarities to trichotillomania, we find that rates of remission before there were evidence-based treatment (i.e. a type of natural recovery) range from 20-24% (Pollitt, 1957; Skoog and Skoog, 1999) (keeping in mind that the diagnosis of “obsessional neurosis from many years ago may not track completely with the current DSM diagnosis of obsessive compulsive disorder). In the case of substance use disorders and gambling disorder, both of which may have some relationship to trichotillomania if it is conceptualized as a behavioral addiction, research suggests that many of those who recover are able to do so without any formal treatment. The rate of natural recovery among individuals with alcohol use disorders has ranged from 24.4% to 78% (Bischof et al., 2005; Dawson, 1996; Dawson et al., 2005; Sobell et al., 1996) (the range may be due to remission being defined by either abstinence or moderate drinking without meeting criteria for an alcohol use disorder) (Mellor et al., 2019). In the related area of gambling addiction, two large national U.S. surveys found that 33%–36% of people with gambling disorder experienced natural recovery (Slutske, 2006). Another study found that around 75% of young adults with subsyndromal disordered gambling at baseline no longer had such symptoms at 1-year follow up (Grant et al., 2014).

Some predictors of natural recovery in the alcohol field include being female, older age, and married; however, severity of alcoholism was negatively correlated with chance of natural recovery (Dawson et al., 2005). In the area of gambling disorder, being female appears to be associated with a greater likelihood of natural recovery (Slutske et al., 2009). Natural recovery from sub-syndromal disordered gambling has been associated with lower amounts spent gambling at baseline, and with older age, albeit within the context of young adults followed up for 1-year (Grant et al., 2014). There is some indication in the obsessive compulsive disorder literature that older age of disorder onset was associated with greater likelihood of natural recovery (Skoog and Skoog, 1999).

Based on this background, the aims of the present study were to document the rates of natural recovery among individuals with trichotillomania and to determine clinical and demographic variables associated with natural recovery.

## Material And Methods

### Participants and Procedures

Data were collected in the context of market research for a client exploring a potential new treatment for trichotillomania. These market research data were then made available in anonymized form to the current researchers, without restriction. The current paper therefore comprises secondary analyses of de-identified data and was thus exempted from Institutional Review Board (IRB) procedures under current US guidelines. All participants had provided informed consent and had agreed that their data could be shared in anonymized form with external researchers.

A convenience sample of approximately 10,000 individuals representative of the general US population, 18-69 years of age, were screened for trichotillomania. Survey respondents were recruited from the Schlesinger Group, an ESOMAR (European Society for Opinion and Marketing Research) member that adheres to a globally recognized code of conduct, the jointly developed ICC/ESOMAR Code, for the purposes of such marketing research. Survey respondents were recruited using the “General Population” panel, a well-known provider of panels for online surveys. Quotas were used to obtain a sample that was age and gender matched to the general US Population.

Quality control procedures included: double opt-in; confirmation using photo ID validation (manual) at time of registration for panel; relevant ID and a programming (CAPTCHA) at registration to deter bots; a Red Herring survey to catch people outside of US, hidden questions in registration to catch bots, database checks to identify batches of similar email structure entering panel in short time period, profile checks to identify unlikely combinations of or too many combinations of ailments, and profile checks to identify selection of aberrant choices at different questions at registration and over time on the panel. Participants received 300 points for participation, which had a monetary value of $3.00. The total duration of the survey was approximately 15 minutes.

### Assessments

Each individual completed a self-administered survey via the Internet, which comprised two segments. Part 1: Screening for prevalence: demographics and diagnosis of trichotillomania and comorbidities; and Part 2: Survey of people with current trichotillomania: survey of diagnosis, severity, and life impact. Part 1 of the survey asked about multiple psychiatric disorders with one question (“Please indicate whether you currently have or have ever had any of the following medical conditions”). Trichotillomania (hair-pulling disorder) was one of the listed conditions. All participants indicated either: “Never”, “In the past, but not currently”, or “Currently”. The general question was then followed by specific questions regarding who diagnosed the condition, age of onset, and treatment history. In addition, Part 2 of the survey asked about each of the following diagnostic criteria for trichotillomania: “Repeated pulling of my hair causing hair loss; repeated attempts to stop or decrease the hair pulling; the hair pulling is/was causing me personal distress or causing difficulty in areas of my life; realizing that the hair pulling, or hair loss was not related to some other medical problem or a skin condition; and the hair pulling was not done to try to improve my appearance or what I saw as a flaw.” It also asked each person if they had ever received psychotherapy or prescription medication for trichotillomania; and how severe their symptoms were ‘at their worst’ (mild, moderate, or severe). Only if the person answered affirmatively to hair-pulling/trichotillomania in the list of medical conditions were they then prompted to answer Part 2. “Natural recovery” was defined as an individual reporting they had experienced trichotillomania in the past but not currently; and that they had not received therapy or prescription medication for trichotillomania. The survey data were collected in January, 2019. The advertisements about the survey were designed to be neutrally worded; i.e. did not mention the purpose of the survey, no mention of “health”, nor of any diagnosis, in order to reduce participation bias.

### Data Analysis

Potential differences in (1) demographic and clinical variables and (2) lifetime comorbidities, between those with natural recovery from trichotillomania, and those with current trichotillomania, were identified using analysis of variance (ANOVA) or Likelihood-Ratio (LR) chi-square tests as appropriate. Additional analyses using binary logistic regression were used to control for potential confounding differences between the groups initially identified. Statistical analyses were conducted using JMP Pro Software. Significance was defined as p<0.05.

## Results

The total sample size was 10,169 adults, and this sample mirrored closely key demographic characteristics of the US population (Grant et al., 2020). In total, of 253 participants with lifetime trichotillomania, 78 participants (30.8%) reported that trichotillomania was a past but not current problem. Of the 78 participants who reported lifetime but not current trichotillomania, 63 said they stopped pulling their hair without treatment and 15 reported no longer pulling due to treatment. Therefore, the rate of natural recovery in the entire sample of people with lifetime trichotillomania was 24.9% (63/253).

Demographic and clinical characteristics for those with natural recovery from trichotillomania (n=63), versus those with current trichotillomania (n=175), are shown in Table 1. Those who reported natural recovery were significantly more likely to be older. The two groups did not differ significantly on other demographic variables or on severity of trichotillomania symptoms. For those participants who reported natural recovery, their hair pulling stopped on average after 10.0 years (median 6 years).

The rates of co-occurring lifetime disorders for the two groups are presented in Table 2. After controlling for age, those who reported natural recovery were significantly less likely to report histories of ADHD, OCD, panic disorder, skin picking disorder, and tic disorder. These findings were also significant without controlling for age. Lower rates of certain other disorders (anxiety disorder, bipolar disorder, eating disorder) were observed in the initial analyses, in those with natural recovery versus current trichotillomania, but these results were no longer significant once age was controlled for.

## Discussion

This study, the first to examine natural recovery in TTM using an overall sample that was epidemiologically representative of the US general population, found that among adults with a history of the disorder, 24.9% reported that their trichotillomania remitted without any formal psychological or pharmacological treatment interventions. Interestingly, this rate is not dissimilar to natural recovery for obsessive-compulsive disorder, or substance or gambling addiction, as reported in much of that literature (Pollitt, 1957; Skoog and Skoog, 1999; Bischof et al., 2005; Sobell et al., 1996; Dawson, 1996; Dawson et al., 2005; Slutske, 2006). The rate found here is however higher than reported in depression (Mekonen et al., 2021). The finding that roughly one-fourth of adults with a history of trichotillomania recover from their problems suggests that trichotillomania does not always follow a chronic or persisting course, and that different individuals experience a very different course.

In addition, the findings strongly suggest that the lack of comorbidity may have the strongest influence in natural recovery as those who experienced it generally had lower rates of several disorders. The strongest predictor of natural recovery, of the variables examined, was the lack of OCD comorbidity. What actually explains the influence of comorbidity in natural recovery? There are several possible, and non-mutually exclusive, explanations. First, it is possible that trichotillomania with OCD, for example, is different neurobiologically from pure trichotillomania and has a different course of illness. Second, the effects of related comorbidities, such as OCD, may simply make it more difficult for trichotillomania symptoms to improve spontaneously as OCD symptoms (or other traits associated with OCD, such as perfectionism or cognitive rigidity) may reinforce the trichotillomania symptoms (e.g., symmetry obsessions drive pulling hair to even it out). The finding of a fairly high rate of natural recovery needs to be understood in the context of fairly low rates of seeking treatment for trichotillomania. In fact, one study found that less than 20% of individuals with trichotillomania received psychotherapy for their pulling, despite its status as the first-line treatment approach (Woods et al., 2006). Another study found that only 40% of people with trichotillomania received any mental health care specific to trichotillomania (Cohen et al., 1995). This fairly low rate of treatment-seeking is likely due to personal factors (e.g. embarrassment) as well as external barriers (e.g., lack of knowledgeable clinicians) to obtaining help (Woods et al., 2006). The low rate of seeking treatment for trichotillomania is likely due to multiple reasons but one possible explanation, derived from the current study, is that perhaps some people are able to employ strategies themselves that are effective. Thus, evidence from natural recovery could potentially inform formal approaches for treating trichotillomania. For example, one obvious question is whether those who have overcome their trichotillomania on their own did so by completely abstaining from any pulling or whether they were able to continue to pull to some degree without problems. Most of what is known regarding trichotillomania has been garnered from observations in clinical settings when patients were receiving treatment. The results of this study suggest that clinically recruited samples are probably not ideal for some research purposes because findings may not generalize to people in the wider community with the condition. Studies involving people who have recovered from trichotillomania may inform and expand our understanding of the disorder.

It is important to note that the demographic characteristics of the current sample of people with trichotillomania are likely to differ in important ways those of prior research studies. Much prior research into the potential prevalence of trichotillomania used college student convenience samples, which by definition would be younger and with a narrower age range than the current dataset, and would be less representative of the general population than those in the current study. Furthermore, clinical trial studies have tended to include relatively high proportions of women compared to men, and would have restricted participation based on extensive inclusion/exclusion criteria; whereas the current study had roughly equal proportions of male and female individuals with trichotillomania, and did not have such restrictions on study participation. Thus, it is important to consider that natural remission may be different to the rate reported here in particular subgroups of patients, such as those taking part in clinical trials.

This study has several positive features, notably that it is the first large study of trichotillomania examining rates and features of natural recovery. Several limitations, however, should be considered. First, the study was a survey and as such no direct in person interviews were performed. The gold standard for diagnosis is of course clinical interview by a healthcare professional. Due to the bespoke convenience nature of the survey, it did not use gold-standard rating tools but rather pragmatic questions about whether trichotillomania had been diagnosed; and also about whether the different diagnostic criteria were met, from the individual’s perspective. Second, the survey used a non-probability sample. Although the sample demographics paralleled national demographics, it still raises the possibility of selection biases based on personality factors, etc. Third, data on comorbidities are also per participant report and as such may have over- or under- reported specific conditions. Fourth, natural recovery has no agreed upon definition in the field of trichotillomania. Is it enough not to meet DSM diagnostic criteria of should complete abstinence of pulling be the standard? Is a person recovered after one year? Or is more time needed?

In summary, this study examined the rate of natural recovery in trichotillomania in a large representative sample of adults in the USA. Overall, natural recovery in trichotillomania was relatively common (24.9%), and was associated with lower rates of comorbidities of several related disorders, especially (but not exclusively) OCD. These results provide new information for people with trichotillomania in terms of natural recovery rates, and also have clinical implications, serving to highlight the importance of screening for these often over-looked related comorbidities in people with trichotillomania. We hope future studies may examine this important issue and include variables not examined here such as depression and anxiety symptoms, self-esteem, and psychosocial functioning to mention only a few that would deepen our understanding of who may experience natural recovery from trichotillomania.

## Figures and Tables

**Table 1:** Comparison of key demographic and cinical features of the two trichotillomania groups.

	Current Trichotillomania (N=175)	Naturally Remitted Trichotillomania (N=63)	F	p
Age, years	36.0 (12.8)	42.4 (16.3)	10.0223	0.002
Gender, female, N [%]	83 [47.4%]	31 [49.2%]	5.159 LR	0.3067
Racial-ethnic group, White Caucasian, N [%]	127 [72.6%]	47 [74.6%]	0.098 LR	0.7542
Age at onset, years	17.7 (10.8)	15.9 (10.5)	1.0409	0.3089
Reported severity of trichotillomania (at worst), N [%]			2.481 LR	0.2892
Mild	57 [32.6%]	27 [42.9%]		
Moderate	106 [60.6%]	31 [49.2%]		
Severe	12 [6.9%]	5 [7.9%]		
Household income, N [%]			0.892 LR	0.9708
<$25,000	32 [18.3%]	12 [19.1%]		
$25,000 - $50,000	48 [27.4%]	14 [22.2%]		
$50,001 - $75,000	36 [20.6%]	14 [22.2%[		
$75,001 - $125,000	30 [17.1%]	13 [20.6%]		
>$125,000	23 [13.1%]	8 [12.7%]		
Prefer not to answer	6 [3.4%]	2 [3.2%]		
Current relationship status, N [%]			8.065 LR	0.1527
Single, not in relationship	60 [34.3%]	26 [41.3%]		
Single, in a relationship	26 [14.9%]	11 [17.5%]		
Cohabiting with partner	13 [7.4%]	4 [6.4%]		
Domestic partnership	3 [1.7%]	2 [3.2%]		
Married	72 [41.1%]	17 [27.0%]		
Separated	1 [0.6%]	3 [4.8%]		
Education level, N [%]			5.560 LR	0.1351
High school or less	37 [21.1%]	8 [12.7%]		
At least some college education or above	138 [78.9%]	55 [87.3%]		

LR = Likelihood Ratio chi-square test.

**Table 2:** Lifetime history of other mental disorders in the trichotillomania study groups.

	Current Trichotillomania (N=175)	Naturally Remitted Trichotillomania (N=63)	LR chi-square	p	p value for regression model, controlling for age
ADHD	60 (34.29%)	9 (14.29%)	9.894	0.0017	0.0116
Alcohol or drug abuse	67 (38.29%)	17 (26.98%)	2.666	0.1025	
Anxiety Disorder	134 (76.57%)	40 (63.49%)	3.88	0.0489	0.1258
Bipolar Disorder	46 (26.29%)	9 (14.29%)	4.039	0.0445	0.1309
Depression	127 (72.57%)	43 (68.25%)	0.418	0.5181	
Eating Disorder	61 (34.86%)	13 (20.63%)	4.604	0.0319	0.1267
OCD	76 (43.43%)	12 (19.05%)	12.678	<0.001	0.0023
Panic Disorder	71 (40.57%)	14 (22.22%)	7.152	0.0075	0.0268
PTSD	64 (36.57%)	16 (25.4%)	2.676	0.1019	
Skin picking disorder	60 (34.29%)	11 (17.46%)	6.719	0.001	0.0315
Tic disorder	28 (16%)	2 (3.17%)	8.693	0.0032	0.0362
					

LR = Likelihood Ratio chi-square test; ADHD = attention deficit hyperactivity disorder; OCD = obsessive-compulsive disorder; PTSD = post-traumatic stress disorder
